# Predicting overlapping protein complexes based on core-attachment and a local modularity structure

**DOI:** 10.1186/s12859-018-2309-9

**Published:** 2018-08-22

**Authors:** Rongquan Wang, Guixia Liu, Caixia Wang, Lingtao Su, Liyan Sun

**Affiliations:** 1 College of Computer Science and Technology, Jilin University, No. 2699 Qianjin Street, Changchun, 130012 China; 2 Key Laboratory of Symbolic Computation and Knowledge Engineering of Ministry of Education, Jilin University, No. 2699 Qianjin Street, Changchun, 130012 China; 3 School of International Economics, China Foreign Affairs University, 24 Zhanlanguan Road, Xicheng District, Beijing, 100037 China

**Keywords:** Protein-protein interaction networks, Protein complex, Overlapping node, Seed-extension paradigm, Core-attachment and local modularity structure, Node betweenness

## Abstract

**Background:**

In recent decades, detecting protein complexes (PCs) from protein-protein interaction networks (PPINs) has been an active area of research. There are a large number of excellent graph clustering methods that work very well for identifying PCs. However, most of existing methods usually overlook the inherent core-attachment organization of PCs. Therefore, these methods have three major limitations we should concern. Firstly, many methods have ignored the importance of selecting seed, especially without considering the impact of overlapping nodes as seed nodes. Thus, there may be false predictions. Secondly, PCs are generally supposed to be dense subgraphs. However, the subgraphs with high local modularity structure usually correspond to PCs. Thirdly, a number of available methods lack handling noise mechanism, and miss some peripheral proteins. In summary, all these challenging issues are very important for predicting more biological overlapping PCs.

**Results:**

In this paper, to overcome these weaknesses, we propose a clustering method by core-attachment and local modularity structure, named CALM, to detect overlapping PCs from weighted PPINs with noises. Firstly, we identify overlapping nodes and seed nodes. Secondly, for a node, we calculate the support function between a node and a cluster. In CALM, a cluster which initially consists of only a seed node, is extended by adding its direct neighboring nodes recursively according to the support function, until this cluster forms a locally optimal modularity subgraph. Thirdly, we repeat this process for the remaining seed nodes. Finally, merging and removing procedures are carried out to obtain final predicted clusters. The experimental results show that CALM outperforms other classical methods, and achieves ideal overall performance. Furthermore, CALM can match more complexes with a higher accuracy and provide a better one-to-one mapping with reference complexes in all test datasets. Additionally, CALM is robust against the high rate of noise PPIN.

**Conclusions:**

By considering core-attachment and local modularity structure, CALM could detect PCs much more effectively than some representative methods. In short, CALM could potentially identify previous undiscovered overlapping PCs with various density and high modularity.

**Electronic supplementary material:**

The online version of this article (10.1186/s12859-018-2309-9) contains supplementary material, which is available to authorized users.

## Background

Protein complexes are a group of proteins that interact with each other at the same time and space [1]. Identifying PCs is highly important for the understanding and elucidation of cell activities and biological functions in the post-genomic era. However, the identification of PCs based on experimental methods is usually costly and time-consuming. Fortunately, with the development of high-throughput experimental techniques, an increasing number of PPINs have been generated. It is more convenient to mine PCs from PPINs. Thus, computational methods are used to detect PCs from PPINs. Generally, PPINs are represented as undirected graphs, and thus the problem of identifying PCs is usually considered as a graph clustering problem. Recently, many graph clustering methods have been proposed to predict PCs.

### Related work

In this study, we divide graph clustering methods into two categories: hard clustering methods and soft clustering methods. Hard clustering methods produce non-overlapping predicted clusters, and soft clustering methods produce overlapping predicted clusters. Hard clustering methods include the Markov cluster (MCL) [2], restricted neighborhood search clustering (RNSC) [3], Girvan and Newman (G-N) [4], and a speed and performance in clustering (SPICi) [5] methods. Gavin et al. [6] showed that many PCs share some “module” in PPINs. However, these hard cluster methods can only predict non-overlapping clusters. In fact, according to the CYC2008 hand-curated yeast protein complex dataset [7], 207 of 1628 proteins are shared by two or more protein complexes. This shows that some PCs have highly overlapping regions [6, 8, 9]. As a result, some soft clustering methods have been developed to discover overlapping PCs from PPINs, and these soft cluster methods further could be roughly divided into three categories.

The first category is the mining clique methods, which includes CFinder [10], clique percolation method (CPM) [11], and clustering based on maximal cliques (CMC) [12]. These methods aim to extract maximal cliques [13] or near-cliques from PPINs because maximal cliques and near-cliques are considered as potential PCs. Nevertheless, finding all cliques is a NP-complete problem from PPINs and is therefore computationally infeasible. Furthermore, the requirement that a protein complex is always taken as a maximal clique or near-clique is highly restrictive.

The second category is the dense graph clustering methods. To overcome the relatively high stringency, majority of researchers focus on identifying densely connected subgraphs by either optimizing an objective density function or using a density threshold. Some typical methods, such as molecular complex detection (MCODE) [14], repeated random walks (RRW) [15], DPClus [16] and IPCA [17], and CPredictor2.0 [18], etc. Liu et al. studied a set of 305 PCs, which consists of MIPS [19], CYC2008 [7] and Aloy [20], and found that for 40% of PCs, the density is less than 0.5 [20]. Furthermore, although the density function provides a good measurement for the prediction of complexes, and its results depend on cluster size. For example, the density of a cluster containing three proteins is 1.0, whereas the density of a cluster with eight proteins could be 0.45. Therefore, these methods discard many low-density protein complexes. Meanwhile, PPINs with noise (high false positive rate and high false negative rate) are produced by high-throughput experiments. Due to the limitations of the associated experimental techniques and the dynamic nature of protein interaction maps, the dense graph clustering methods are sensitive to noisy data.

The third category is the heuristic graph clustering methods. In recent years, some researchers have attempted to detect PCs by using methods in relevant fields. For examples, PEWCC [21], GACluster [22], ProRank [23], and clustering with overlapping neighborhood expansion (ClusterONE) [24], and they are representative methods for this category. From the standpoint of the results, the heuristic graph clustering methods are effective for the identification of PCs. However, these methods neglect a lot of peripheral proteins that connect to the core protein clusters with few edges [25]. Thus, it is clear that different proteins are different importance for different PCs [26, 27]. Moreover, some heuristic methods are more sensitive to the selection of parameters.

In addition to the abovementioned methods, some existing methods combine different kinds of biological informations to predict PCs. These biological informations include functional homogeneity [28], functional annotations [18, 29, 30], functional orthology information [31], gene expression data [32, 33] and core-attachment structure[33–35]. Although various types of additional biological informations may be helpful for the detection of PCs, the current knowledge and technique for PC detection are limited and incomplete.

### Our work

**Fig. 1:**
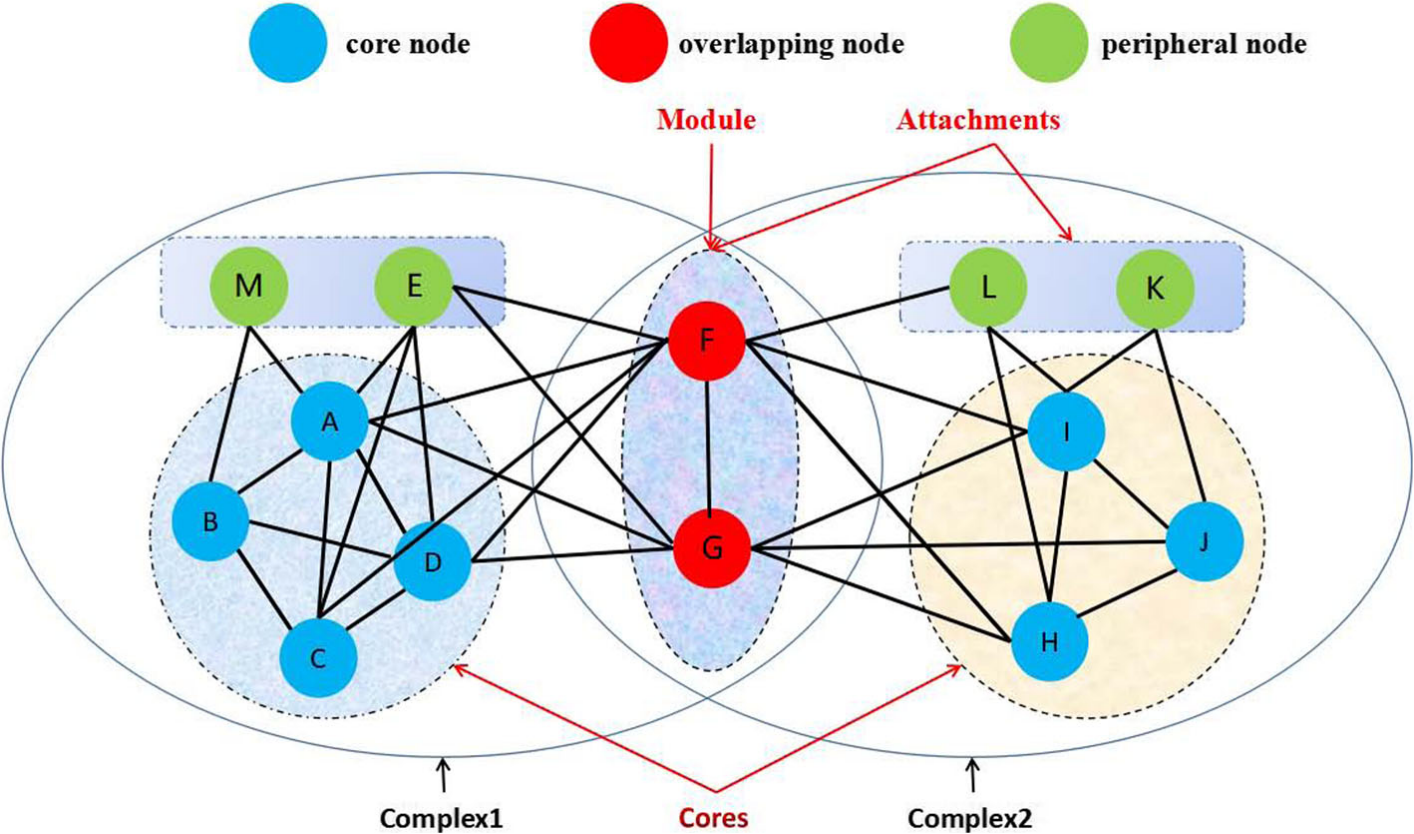
Definition and terminology are used to define overlapping PCs architecture. An example of overlapping PCs, whose core components consist of core nodes in the dashed circle. A PC consists of core components and attachments. Additionally, attachments consist of modules and some peripheral nodes. Note that among the attachments, a “module” is composed of overlapping nodes, and the rest of nodes are called peripheral node. The three types of nodes are marked by different colors. Two overlapping PCs are circled by solid lines

Although previous methods can effectively predict the PCs from PPINs, the internal organizational structure of the PCs is usually ignored. Some researchers have found that the PCs consist of core components and attachments [6]. Note that Core components are a small group of core proteins that connect with each other, and have high functional similarity. Core components play a significant role in the core functions of the complex and largely determine its cellular function. Meanwhile, attachments consist of modules and some peripheral proteins. Among the attachments, there are two or more proteins are always together and present in multiple complexes, which authors call “module” [6, 9], for examples, the overlapping nodes F and G in Fig. 1 consist of a module. In this paper, we consider the PCs have core-attachment and local modularity structure. Local modularity means that the PCs have more internal weighted than external weighted connections. Figure 1 shows the model of overlapping PC structure.

CALM method is based on the seed-extension paradigm. Therefore, CALM mostly focuses on the following two aspects: the selection of the seed nodes and CALM starts from a seed node and continuously check its neighboring nodes to expand the cluster. In this work, on the one hand, according to core-attachment structure, the consideration of core nodes as seed nodes to predict complexes is very important, and by contrast many current methods simply select seed nodes through their degree and correlative concepts. Because of this, they could not distinguish between core nodes and overlapping nodes. As a result, these methods mistake and miss a number of highly overlapping PCs. For instance, two highly overlapping PCs may be identified as a fake complex, whereas they are actually functional modules. Our findings suggest that node betweenness and node degree are two good topology characters to distinguish between the core nodes and overlapping nodes. On the other hand, PCs tend to show local modularity with dense and reliable internal connections and clear separation from the rest of the network. Thus, we use a local modularity model incorporating a noise handling strategy to assess the quality of the predicted cluster. Furthermore, we design a support function to expand the cluster by adding neighboring nodes.

The experimental results have shown that CALM could predict overlapping and varying density PCs from weighted PPINs. Three popular yeast PPI weighted networks are used to validate the performance of CALM, and the predicted results are benchmarked using two reference sets of PCs, termed NewMIPS [36] and CYC2008 [7], respectively. Comparison to ten state-of-the-art representative methods, the results show that the CALM outperforms some computational outstanding methods.

## Methods

In this section, we will introduce some basic preliminaries and concepts at first. We then describe the CALM algorithm in the following subsections.

### Preliminaries and concept

Mathematically, a PPI network is often modeled as an undirected edge-weighted graph *G*=(*V*,*E*,*W*), where *V* is the set of nodes (proteins), *E*={(*u*,*v*)|*u*,*v*∈*V*} is the set of edges (interactions between pairs of proteins), and *W*:*E*→ℜ^+^ is a mapping from an edge in *E* to a reliable weight in the interval [0,1].

**Fig. 2:**
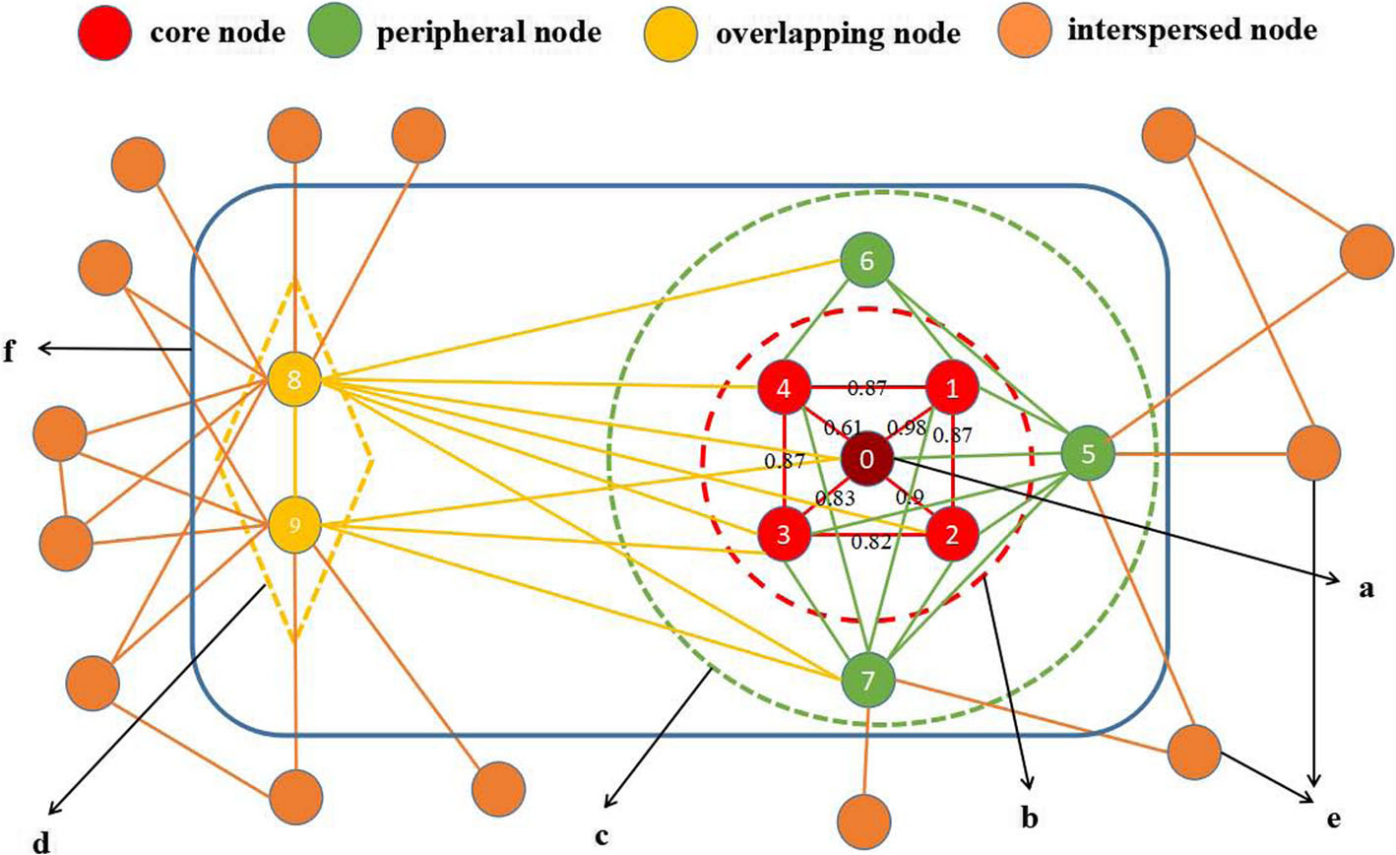
The formation process of a protein complex. The four type of nodes are marked by different colors. **a** the deep red protein represents the seed protein; **b** these red proteins inside the red dotted circle constitute a complex core; **c** these green proteins inside the green dotted circle represent peripheral proteins; **d** the yellow proteins inside the yellow dotted circle represent overlapping proteins; **e** the chocolate yellow proteins represent interspersed node; **f** complex core, peripheral proteins, and overlapping proteins inside the blue circle constitute a protein complex; An example illustrates the clustering process. This simple network has 22 nodes, and each edge has weight 0.2 except (0,1),(0,2),..., and (3,4). The node 0 is taken as a seed protein and the initial cluster {0} is constructed. In the greedy search process, the neighbors of the node 0 include {1,2,3,4,5,8,9}. The node 1 has the highest support function $\frac {0.98}{0.98+0.87+0.87+0.2*3}=0.295$ according to support function (Eq. (7)). We add node 1 to the cluster, and if the value of local modularity score increases, then this cluster is {0,1}. Similarly, the nodes 2, 3, and 4 are added to the cluster in sequence and now the neighbors of node 0 include 5, 8, 9 are left, the node 5 has the highest support function, but when the node 5 is added to the cluster {0,1,2,3,4}, its local modularity score decrease. Thus the node 5 is removed from the cluster and this greedy is terminated. Now the cluster {0,1,2,3,4} constitutes the complex core. We do the next greedy search to extend the complex core to form the whole complex. Furthermore, for the complex core {0,1,2,3,4}, its neighboring nodes have the nodes 5, 6, 7, 8, and 9, we repeat iteration this process for the cluster until the cluster isn’t change and save it as the first cluster. Similar, the next search will start from the next seed node to expand the next cluster

As shown in Fig. 2, using this model for a given PPI weight network, and all the nodes in the PPI network can be classified into four types. First, we consider that a node is a “core node” in a complex if: (a) As described by Gavin et al, it shows the degree of similarity of physical association, high similarity in expression levels, and represents the functional units within the complex; (b) Core nodes display relatively high weighted degree of direct physical interactivity among themselves and less interactions with the nodes outside the complex; (c) Each protein complex has a unique set of core nodes. The second category is “peripheral node”. A node is considered as a peripheral node to a complex if: (a) It interacts closely with the core of the complex and shows greater heterogeneity in expression level. (b) It is stable and directly reliable with complex core. The third category is the “overlapping node”. A node is considered to be an overlapping node to a complex if: (a) It shows a higher degree and node betweenness than its neighboring nodes. (b) It belongs to more than a complex. (c) It interacts closely with the core nodes. All remaining nodes are classified as “interspersed node”, which is likely to be the noise in PPI network.

### Identifying overlapping nodes

Two or more overlapping nodes in static PPI networks always gather together to form “module” which is an indispensable feature that plays important roles at various levels of biological functions. Moreover, overlapping nodes participate in more than one PC. Overlapping nodes are identified in order to prevent their use as seed nodes, which could lead to the result that some high overlapping PCs are wrongly predicted, whereas in fact it is a functional module. Furthermore, it is necessary to explain the differences between the two concepts. Li [37] believes that functional modules are closely related to protein complexes and a functional module may consist of one or multiple protein complexes. Li [37] and Spirin [49] have suggested that protein complexes are groups of proteins interacting with each other at the same time and place. However, functional modules are groups of proteins binding to each other at a different time and place.

To better understand the difference between protein complexes and functional modules, we give a example that Complex1 and Complex2 are protein complexes, but a combination of both Complex1 and Complex2 could constitute a functional module when overlapping nodes such as F or G are used as seed nodes in Fig. 1. In this case, some high overlapping PCs could be mistaken or omitted, and then it may mistakenly predict that Complex1 and Complex2 constitute a predicted PC, and Complex1 and Complex2 may be omitted in some previous methods. Therefore, we need to identify overlapping nodes. In social network analysis, degree and betweenness centrality are commonly used to measure the importance of a node in the network. Here, we find that the degree and betweenness are effective for the identification of overlapping nodes. The degree and node betweenness of overlapping nodes are larger than the average of all their neighboring nodes because overlapping nodes participate in multiple complexes.

For a node *v*∈*V*,*N*(*v*)={*u*∣(*v*,*u*)∈*E*} denotes the set of neighbors of node *v*, *deg*(*v*)=|*N*(*v*)| is the number of the neighbors of node *v*. Given a node *v*∈*V*, its local neighborhood graph *GN*_*v*_=(*V*_*v*_,*E*_*v*_) is the subgraph formed by *v* and all its immediate neighboring nodes with the corresponding interactions in *G*. It can be formally defined as *GN*_*v*_=(*V*_*v*_,*E*_*v*_), where *V*_*v*_ = {*v*}∪{*u*∣*u*∈*V*,(*u*,*v*)∈*E*}, and *E*_*v*_ = {(*u*_*i*_,*u*_*j*_)∣(*u*_*i*_,*u*_*j*_)∈E,*u*_*i*_,*u*_*j*_∈*V*_*v*_}.

We define the average weighted degree of *GN*_*v*_ as *Avdeg*(*GN*_*v*_) and calculate it according to Eq. (1).

1 $$ {Avdeg\left(GN_{v}\right)=\frac{\sum_{u\in V_{v}}deg(u)}{\lvert V_{v}\rvert}}  $$

Theoretically, |*V*_*v*_| represents the number of local neighborhood subgraphs *GN*_*v*_with nodes, and $\sum _{u\in V_{v}}deg(u)$ represents the sum of *deg*(*u*) for all nodes in *V*_*v*_.

The node betweeness, *B*(*v*), is a measure of the global importance of a node *v*, and it can assess the fraction of shortest paths between all node pairs that pass through the node of interest. A more in-depth analysis has been provided by Brandes et al. [38–40]. For a node *v*, its node betweenness (*B*(*v*)) is defined by Eq. (2).

2 $$ { B(v)= \sum_{s\neq v\neq t\in V} \frac{\delta_{s,t}(v)}{\delta_{s,t}} }  $$

Herein, *δ*_*s*,*t*_ is the number of shortest paths from node *s* to *t* and *δ*_*s*,*t*_(*v*) is the number of shortest paths from node *s* to *t* that pass through the node *v*. For each node *v*, the average node betweenness of its local subgraph *GN*_*v*_ is defined as the average of *B*(*u*) for all *u*∈*V*_*v*_ and written as *AvgB*(*GN*_*v*_) in Eq. (3).

3 $$ { AvgB\left(GN_{v}\right) = \frac{\sum_{u \in N_{v}}B(u)}{\lvert V_{v}\rvert } }  $$

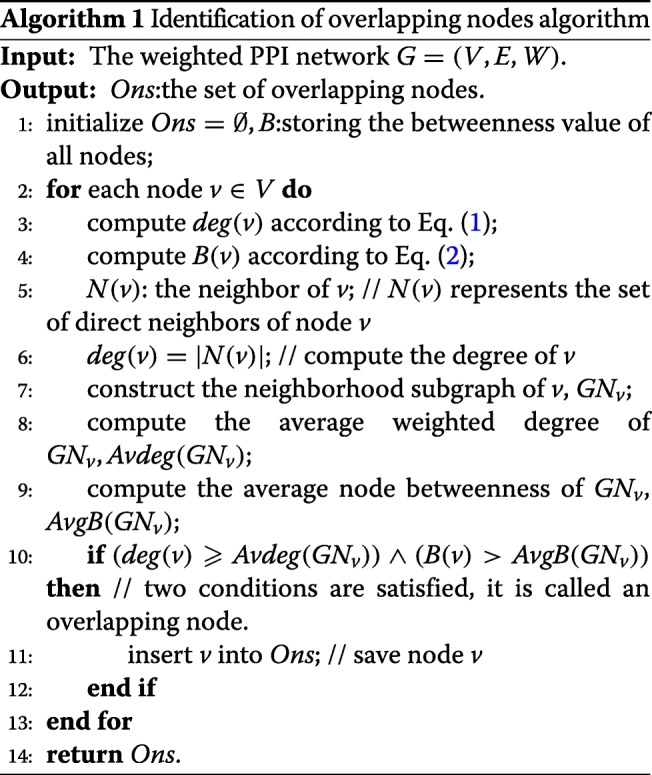


Algorithm 1 illustrates the framework of identifying the overlapping nodes. For each node *v* in the whole PPIN, if the degree of *v* is larger than or equal to *Avgdeg*(*GN*_*v*_), i.e, $deg(v)\geqslant Avdeg\left (GN_{v}\right)$, and the betweenness of *v* is larger than *AvgB*(*GN*_*v*_), i.e, *B*(*v*)>*AvgB*(*GN*_*v*_). If and only if these two conditions are satisfied, the node *v* is classified as an overlapping node in lines 2-13.

### Selecting seed nodes

The strategy for the selection of seed nodes is very important for the identification of PCs. However, most of existing methods are based primarily on node degree for the selection of seed nodes. However, this strategy is too simplistic to detect overlapping PCs. A previous study [41] has observed that the local connectivity of a node plays a crucial role in cellular functions. Therefore, in this paper, we use some topology properties including degree, clustering coefficient and node betweenness to assess the importance of nodes in a PPIN.

Furthermore, Nepusz et al. [24] concluded that network weight can greatly improve the accuracy of identification PCs. Therefore, we use weighted PPINs described in Ref [24] to predict PCs. The definitions of node degree and clustering coefficient could be extended to their corresponding weighted versions as described in Eqs. (4) and (5).

4 $$ { deg_{w}(v)= \sum_{u \in N(v);(v,u)\in E}w_{v,u} }  $$

The small-world phenomenon tends to be internally organized into highly connected clusters and has small characteristic path lengths in biological networks [42–44]. This corresponds to the local weighted clustering coefficient (*LWCC*). The *LWCC*(*v*) of a node *v* could measure its local connectivity among its direct neighbors. The *LWCC*_*w*_(*v*) of a node *v* is the weighted density of the subgraph *GN*_*v*_ formed by *N*_*v*_ and their corresponding weighted edges, and thus we define its *LWCC*_*w*_(*v*) as follows Eq. (5).

5 $$ {LWCC_{w}(v)=\frac{\sum_{i\in V_{v}}\sum_{j\in N(i)\cap V_{v}}w_{i,j}}{\lvert N_{v}\rvert\times (\lvert N_{v}\rvert-1)}}  $$

where $\frac {1}{2}\sum _{i\in V_{v}}\sum _{j\in N(i)\cap V_{v}}w_{i,j}$ is the sum of the weighted degree of subgraph *GN*_*v*_ and |*N*_*v*_|×(|*N*_*v*_|−1)/2 is the maximum number of edges that could pass through node *v*. Note that 0≤*LWCC*≤1. *LWCC*_*w*_(*v*) is not sensitive to noise. Therefore, *LWCC*_*w*_(*v*) is more suitable for the large-scale PPINs which contains many false-positive data.

6 $$ {AvgLWCC_{w}(v)=\frac{\sum_{u\in V_{v}}LWCC_{w}(u)}{\lvert N_{v}\rvert}}  $$

where *LWCC*_*w*_(*v*) is the local weighted clustering coefficient of the node *v*. Note that *N*_*v*_ stands for the number of the node *v* and all its neighbours in local subgraph. Finally, for each node *v*, we compute the average *LWCC*_*w*_(*v*) of subgraph *GN*_*v*_ is denoted as *AvgLWCC*_*w*_(*v*) in Eq. (6).

Central complex members have a low node betweenness and are core nodes (also called hub-nonbottlenecks in [39]). Because of the high connectivity inside complexes, paths can go through them and all their neighbors such as the nodes I, J and H in Fig. 1 according to Eq. (2). On the other hand, overlapping nodes (also called hub-bottlenecks in [39]) tend to correspond to highly central proteins that connect several complexes or are peripheral members of central complexes such as the nodes F and G in Fig. 1 according to Eq. (2) [39, 45]. We check two conditions before a node is considered to be a seed node. First, a node *v* is not an overlapping node, but the *LWCC*_*w*_(*v*) value of *v* in *GN*_*v*_ is still larger than or equal to the average *LWCC*_*w*_(*v*) value of *GN*_*v*_,i.e., $LWCC_{w}(v)\geqslant AvgLWCC_{w}(v)$. Second, we check whether the node betweenness *B*(*v*) of node *v* in *GN*_*v*_ is smaller than the average node betweenness of its neighbor members, i.e., *B*(*v*)≤*AvgB*(*GN*_*v*_). If at least one of two conditions is satisfied, this node is considered as a seed node in lines 2-10. Algorithm 2 illustrates the framework of the seed generation process.


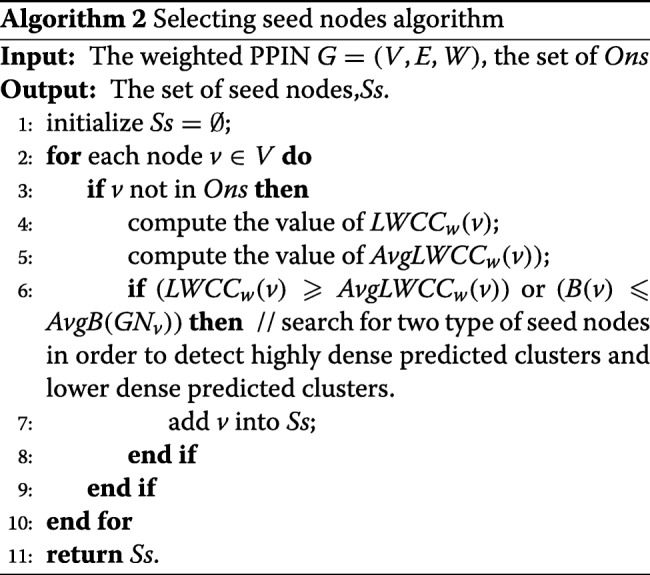


### Introducing two objective functions

In this section, we use two objective functions to solve a seed node is expanded to a cluster. Firstly, the support function is used to determine that the priority of a neighboring node of a cluster. Secondly, local modularity function determines whether a highest priority node is added to a cluster.

#### Support function

A cluster *C*_*p*_ is expanded by gradually adding neighbor nodes according to the measure of similarity strategy. Since we suggest that the higher similarity value a neigibor node *u* has, the more likely it is to be in the cluster *C*_*p*_. Therefore, we introduce the concept of support function to measure how similarly a node *u* with respect to the cluster *C*_*p*_. The task of support function is to eliminate errors when adding a node to a cluster and avoid some peripheral proteins such as node 6 in Fig. 2 are missed. The *support*(*u*,*C*_*p*_) of a node *u* is connected to the cluster *C*_*p*_ is defined as Eq. (7).

7 $$ {support\left(u,C_{p}\right)=\frac{\sum_{v\in C_{p}\cap N(u)}w_{u,v}}{\sum_{v\in N(u)}w_{u,v}}}  $$

where *u*∉*C*_*p*_, and $\sum _{v\in C_{p}\cap N(u)}w_{u,v}$ is the sum of the weight edges connecting the node *u* and *C*_*p*_, and $\sum _{v\in N(u)}w_{u,v}$ is the sum of weights degree the node *u*. Obviously, it takes a value from 0 to 1.

We use an example to make some statements more clearly. As shown in Fig. 2, the blue circle is a protein complex, named *C*_*p*_. Supposing node 0 is a seed node, and for its a neighboring node, its support function is calculated according to Eq. (7). On the one hand, a core node directly connects with all nodes in *C*_*p*_. For the node 1, all its neighbors are in *C*_*p*_, thus the support function of the node 1 is 1.0. Moreover, these red proteins inside the red dotted circle constitute a complex core. On the other hand, a peripheral node could connect to some nodes in *C*_*p*_. For instance, the number of neighbors for node 5 is 9. However, it connects to the node 0, 1, 2, 3, 6, and 7, and its support function is $\frac {6\times 0.2}{9\times 0.2}=\frac {2}{3}$. Finally, an overlapping node has higher degree because it has many neighbors. However its support function is very low. For instance, for node 8, its support function is $\frac {6\times 0.2}{13\times 0.2}=\frac {6}{13}$. In this case, the support function of the nodes 1, 5, and 8 are 1.0, $\frac {2}{3}$, and $\frac {6}{13}$. It is obvious that core nodes and peripheral nodes have priority over overlapping nodes when a node is inserted into the cluster *C*_*p*_.

The support function is very different from Wu et al. [34]’s *closeness*(*v*,*C*). Wu et al.’s measure could only detect the attachment proteins which are closely connected to the complex core such as the nodes 5 and node 7 in Fig. 2. But some attachment proteins that may connect to the complex core with few edges even though its support function is relatively large. This type of attachment proteins, for example, node 6 in Fig. 2, may be missed.

#### Local modularity function

Whether a neighbor node *u* is inserted into a cluster *C*_*p*_ is decided by the local modularity score (*F*(*C*_*p*_)) between *u* and *C*_*p*_. For a clear description, we first provide some relate concepts. In an undirected weighted graph *G*, for a subgraph *C*_*p*_(*C*_*p*_⊆*G*), its weighted in-degree, denoted as *weight*_*in*_(*C*_*p*_), is the sum of weights of the edges connecting node *v* to other nodes in *C*_*p*_, and its weighted out-degree, denoted as *weight*_*out*_(*C*_*p*_), is the sum of weights of edges connecting node *v* to nodes in the rest of *G* (*G*−*C*_*p*_). Both *weight*_*in*_(*C*_*p*_) and *weight*_*out*_(*C*_*p*_) can be defined by Eqs. (7) and (8), respectively.

8 $$ {\kern5pt}{weight_{in}\left(C_{p}\right)=\sum_{v,u\in C_{p},w_{v,u}\in W}w_{v,u}}  $$

9 $$ {weight_{out}\left(C_{p}\right)=\sum_{v\in C_{p},u\notin C_{p},w_{v,u}\in W}w_{v,u}}  $$

In many previous methods, dense subgraphs are considered as PCs. Nevertheless, because real complexes are not always highly dense subgraphs. Many researchers study the topologies of protein complexes in PPINs and find that PCs exhibit a local modularity structure. Meanwhile, we also take into account the core-attachment structure. Generally, a local modularity of subgraph in a PPIN is defined as the sum of weighted in-degree of all its nodes, divided by the sum of the weighted degree of all its nodes. Based on these structural properties, we have improved a local modularity function based on a fitness function [24, 46, 47]. This function has a noise handing strategy, which makes it insensitive to noise in PPINs. The subgraph of local modularity [46, 48] is defined by Eq. (10).

10 $$ {} \begin{aligned}{F\left(C_{p}\right)\,=\,\frac{weight_{in}\left(C_{p}\right)}{\left(weight_{in}\left(C_{p}\right)+weight_{out}\left(C_{p}\right)+ \delta*\left|V_{p}\right|\right)^{\alpha}} }\end{aligned}  $$

Obviously, *F*(*C*_*p*_) takes a value from 0 to 1. Here, *δ* is a modular uncertainty correction parameter. In fact, because of the limitation of biological experiments, nodes with false positive and false negative interactions exist in PPINs. Therefore, this parameter is not only a representative of *δ* undiscovered interactions for each node in the cluster but also a measure to mean noise for the cluster. The value of *δ* depends on half of the average node degree in a PPIN under test because most of PPINs have a higher proportion of noisy protein interactions (up to 50%) [49]. Herein, |*V*_*p*_| represents the size of set *C*_*p*_. What’s more, we choose *α*=1 because it is the ratio of the internal edges to the total edges of the community. It corresponds to the so-called weak definition of the community introduced by Radicchi et al. [50]. In summary, we use this local modularity function in order to find a lot of subgraphs with a high *weight*_*in*_(*C*_*p*_) and a low *weight*_*out*_(*C*_*p*_). This model is an easy and efficient to detect the optimal and local modularity cluster.

### Generating candidate clusters

After obtaining all seed nodes and introducing two objective function, we use an iterative greedy search process to grow each seed node. In our work, we use a local modularity function which aims to discover various density and high modularity PCs. In other words, PCs are densely connected internally but are sparsely connected to the rest of the PPI network. Therefore, we use a local modularity function to estimate whether a group of proteins forming a locally optimal cluster.

In Algorithm 3, firstly, we pick first seed node in the queue *Ss* and use it as a seed to grow a new cluster in line 3. At the same time, the selected seed node is removed from *Ss* in line 4, and then we define a variable *t* to record the number of iterations in line 5. Secondly, we try to expand the cluster from the seed node by a greedy process. This greedy growth process is described in lines 6-22. As a demonstration, we use a simple example in Fig. 2 to explain CALM more intuitively.


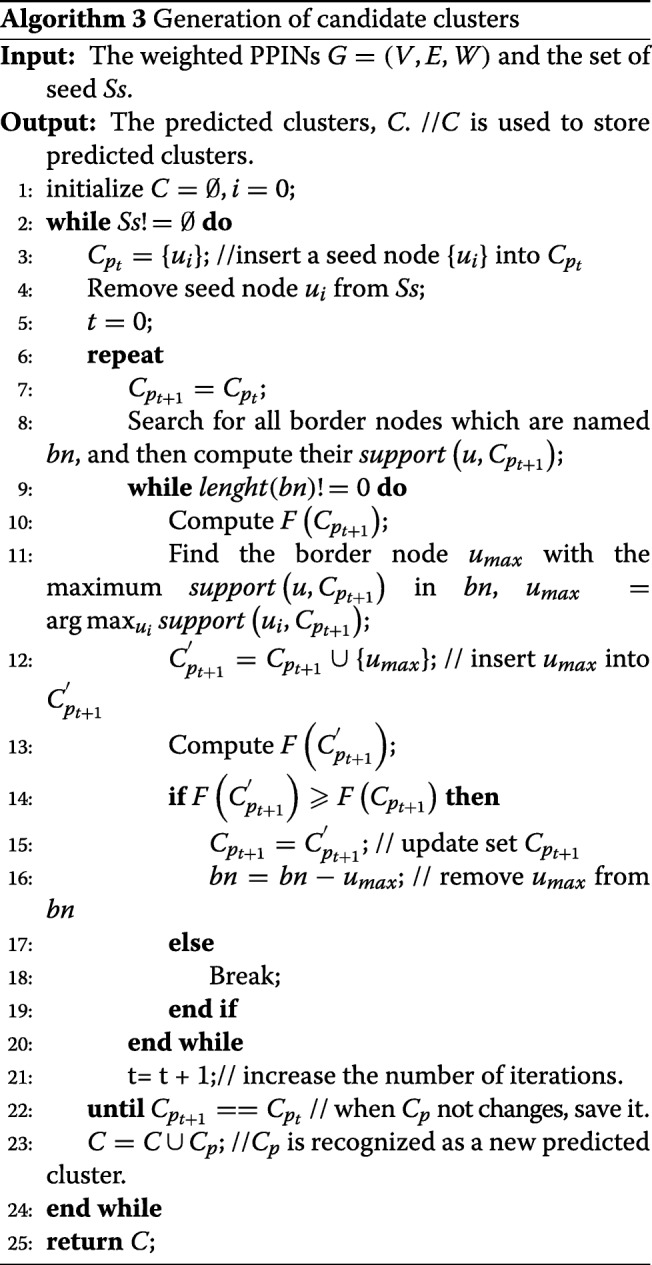


In this process, for the cluster, *C*_*p*_, we first search for all its border nodes that are adjacent to the node in *C*_*p*_ and compute their *support*(*u*,*C*_*p*_) in line 8. Then, we calculate $F\left (C_{p_{t+1}}\right)$, and find the border node with having the maximum *support*(*u*,*C*_*p*_) among all border nodes, named *u*_*max*_ in lines 10-11. Meanwhile, we calculate $F\left (C^{'}_{p_{t+1}}\right)$ when *u*_*max*_ is inserted into $C_{p_{t+1}}$ in lines 12-13. If $F\left (C^{'}_{p_{t+1}}\right)\geqslant F\left (C_{p_{t+1}}\right)$, it means that the local modularity score increases in line 14. *u*_*max*_ should be added to the cluster $C_{p_{t+1}}$, and $C_{p_{t+1}}$ is updated, i.e in line 15. Additionally, *u*_*max*_ is removed from the set of border nodes *bn*. We iteratively add the border node with having maximum *support*(*u*,*C*_*p*+1_) until the set of border nodes is null in line 9 or the local modularity score does not increase in line 18, otherwise this growth process finishes. Then we let *t*=*t*+1 to do the next iteration in lines 7-21, the current cluster’s all border nodes are re-researched and their support functions are re-computed in line 8, and this greedy process is repeated for the cluster until the cluster does not change in lines 6-22. *C*_*p*_ is considered as a new candidate cluster in line 23. The entire generation of candidate clusters processes terminates when the seed set *Ss* is null in line 24. At last, we return all candidate clusters *C* in line 25. Algorithm 3 illustrates overall framework for the generation of candidate clusters.

### Merging and removing some candidate clusters

In Algorithm 4, CALM removes and merges highly overlapped candidate clusters as follows. For each candidate cluster *C*_*i*_ in lines 1-8, CALM checks whether there exists a candidate cluster *C*_*j*_ such that $OS\left (C_{i},C_{j}\right)\geqslant \omega $ in lines 2-3. If such *C*_*j*_ exists, then *C*_*j*_ is merged with *C*_*i*_ in line 4, and simultaneously *C*_*j*_ is removed in line 5. Here, *OS*(*C*_*i*_,*C*_*j*_) is calculated according to Eq. (11), and merge threshold *ω* is a predefined threshold for merging.

11 $$ {OS(A,B)=\frac{\arrowvert A \cap B\arrowvert^{2}}{\arrowvert A \arrowvert \times \arrowvert B\arrowvert}}  $$

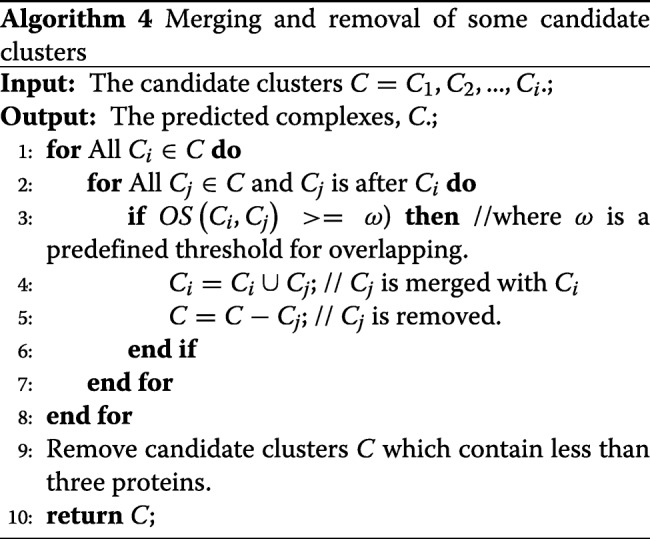


In this paper, we set *ω* is 1 (see “Parametric selection” section). It means that if there are two identical candidate clusters, only one cluster is kept. Furthermore, we remove the candidate clusters with the size less than 3 in line 9 because these candidate clusters could be easily considered as real complexes, which may give rise to randomness in the final result and affect the correctness of the performance evaluation. For instance, that the size of a complex is 2: $OS = \frac {1}{(2*2)}=0.25 >0.2$ can be considered a protein complex. Algorithm 4 shows the pseudo-codes of merging and removal of candidate clusters.

### CALM is different from ClusterONE

In this section, we provide a summary of the ClusterONE of Nepusz et al. [24] and show how CALM differs from ClusterONE.

- We have fully considered the inherent core-attachment organization of PCs in CALM, but ClusterONE had not taken account of this structure. It is the biggest difference between CALM and ClusterONE. (see “Our work” section)
- Though researchers believed that it is very important to distinguish between overlapping nodes and seed nodes, they did not distinguish between the two because existing clustering algorithms lacked some topological properties in the analyzed PPI networks. However, the CALM first provides an approach to distinguish them, because it is very important to predict overlapping protein complexes. (see “Identifying overlapping nodes” section)
- ClusterONE selects the next seed by considering all the proteins that have not been included in any of the protein complexes found so far and taking the one with the highest degree again. ClusterONE ignores a basic fact that overlapping nodes could belong to multiple complexes according to overlapping nodes have higher degree, and overlapping nodes are considered as seed nodes, which can lead to some high overlapping protein complexes being wrongly considered as a single fake PC (In fact, they are functional modules) or miss some high overlapping protein complexes. The influence of this effect has been illustrated in the “Identifying overlapping nodes” section.
- We propose the support function could eliminate errors when adding a node to a cluster and avoid some peripheral proteins are missed. The support function has two important functions. First, one is that it could eliminate errors. Second, it could avoid some peripheral proteins are missed. (see “Support function” section)
- For ClusterONE, we think that it is too strict to make the “cohesiveness” be larger than a threshold (1/3), because some protein complexes have a lower threshold (their “cohesiveness” may be smaller than 1/3), and they could be missed. Therefore, it is more reasonable to let a predicted cluster become a locally optimal modularity cluster. (see “Generating candidate clusters” section)
- ClusterONE extends a cluster (starting with a highest degree seed) by alternately adding and deleting some nodes to make “cohesiveness” satisfy a threshold. Our method adds nodes greedily by the support function to make local modularity function reach local optimal cluster. Moreover, ClusterONE sets *p* to default 2. In this paper, the value of *δ* is half of the average node degree in a entire PPIN. Therefore, CALM is more adaptable to different networks. (see “Local modularity function” section)

## Results and discussion

### Datasets

We use three large-scale PPINs of saccharomyces cerevisiae of Collins et al. [51], Gavin et al. [6] and Krogan et al. [52] to test the CALM method, and they are also used in ClusterONE [24]. These PPINs are assigned a weight representing its reliability thought derived from multiple heterogeneous data sources. For Collins et al. [51], we use the top 9,074 interactions according to their purification enrichment score. The Gavin et al. [6] are obtained by considering all PPINs with a socio-affinity index larger than 5. The Krogan et al. [52] uses a variant: Krogan core contained only highly reliable interactions (probability >0.273). Self-interactions and isolated proteins are eliminated from these datasets. The properties of the three PPINs used in the experimental work are shown in Table 1.

**Table 1:** The properties of the three datasets used in the experimental study

Dataset	Proteins	Interactions	Network density	Average no.of neighbors
Collins	1622	9074	0.007	11.189
Gavin	1855	7119	0.004	8.268
Krogan core	2708	7123	0.002	5.261

Table 2 gives two sets of reference PCs, which are used as gold standards to validate the predicted clusters. The first benchmark dataset is the CYC2008 which consists of manually curated PCs from Wodark’s lab [7]. The second benchmark dataset is derived from three sources: MIPS [19], Aloy et al. [20] and the Gene Ontology(GO) annotations in the SGD database [53]. Complexes with fewer than 3 proteins are filtered from two benchmarks. There are 236 complexes left in the CYC2008 and 328 complexes left in NewMIPS. To illustrate that the real-world PCs are overlapping, we compute the number of overlapping and non-overlapping PCs in the two reference sets. The results are shown in detail in Table 2. It is shown that 86.28% and 45.77% PCs in CYC2008 [7] and NewMIPS [36] are overlapping, respectively. Therefore, to improve the prediction accuracy of graph clustering methods, it is critical that the overlapping problem is solved.

**Table 2:** The statistics of benchmark datasets

Complex dataset	Overlapping complexes	Non-overlapping complexes	The sum of complexes
NewMIPS	283(86.28%)	45(13.72%)	328(100%)
CYC2008	108(45.77%)	128(54.23%)	236(100%)

### Evaluation criteria

To assess the performance by comparison between the predicted clusters and the reference complexes, the most commonly method used is the geometric accuracy (ACC) measure introduced by Brohee and van Helden et al. [54]. This measure is the geometric mean of clustering-wise sensitivity (Sn) and the positive predictive value (PPV). Given *N* complexes as references complexes and *M* predicted complexes, let *t*_*ij*_ represent the number of the proteins in both the reference complex *N*_*i*_ and predicted complex *M*_*j*_. Sn (12), PPV (13), and ACC (14) are defined as follows.

12 $$ {\kern9pt}{S_{n}=\frac{\sum_{i=1}^{n}max_{j=1}^{m}\left\{t_{ij}\right\}}{\sum_{i=1}^{n}N_{i}}}  $$

13 $$ {PPV =\frac{\sum_{j=1}^{n}max_{i=1}^{n}\left\{t_{ij}\right\}}{\sum_{j=1}^{m}\sum_{i=1}^{n}t_{ij}}}  $$

14 $$ {ACC=\sqrt{S_{n}\times PPV}}  $$

Sn measures the fraction of proteins in the reference complexes that are detected by the predicted complexes. Since PPV could be maximized by putting each protein in its own cluster, so it is necessary to balance these two measures by using ACC. It should be noted that ACC can not turn them into a perfect criterion for the evaluation of complex detection methods. This is because the value of PPV can be misleading if some proteins in the reference complex appear in either more than one predicted complex or in none of them. There are substantial overlaps between the predicted complexes, and this puts the overlapping clustering methods at a disadvantage. Therefore, the PPV value is always smaller than the actual value. The geometric accuracy measure explicitly penalizes predicted complexes that do not match any of the reference complexes [24].

Therfore, Nepusz et al. [24] proposed two new measure of the maximum matching ratio (MMR) and fraction criterion to overcome this defect. There is a difference between the basic assumptions of MMR and ACC. The MMR measure reflects how accurately the predicted complexes represent the reference complexes by using maximal matching in a bipartite graph [55] to compute the matching score between each member of the predicted part and each member of the reference part which is computed by the equation (11), and if the calculated value is bigger than 0.25, then a maximum weighted bipartite graph matching method is executed. Therefore we obtain a one-to-one mapping maximal match between the member of two sets. The value of MMR is given by the total weight of the maximum matching, divided by the number of reference complexes. MMR offers a natural and intuitive way to compare the predicted complexes with a gold standard, and it explicitly penalizes cases when a reference complex is split into two or more parts in the predicted set, because only one of its parts is allowed to match the correct reference complex. If *P* denotes the set of predicted complexes and *R* denotes the set of reference complexes, the fraction criterion Eq. (16) is then defined as follows.

15 $$ {\kern23pt}{N_{r}=\arrowvert\lbrace c|c\in R, \exists p \in P,OS(p,r)\geq \omega\rbrace}\arrowvert  $$

16 $$ {Fraction=\frac{N_{r}}{|R|}}  $$

As mentioned below, *OS*(*p*,*r*) is a matching score, which is computed to measure the extent of matching between a reference complex *r* and a predicted complex *p*. Therefore, it represents the fraction of reference complexes, which are matched by at least one predicted cluster. We set this threshold *w* to 0.25, which means at least half of the proteins in the matched reference complexes are the same as at least half of the proteins in the matched predicted cluster. Finally, we compute the sum of the accuracy, MMR and fraction criteria for comparing the performance of the complex detection methods [24].

### Parametric selection

**Fig. 3:**
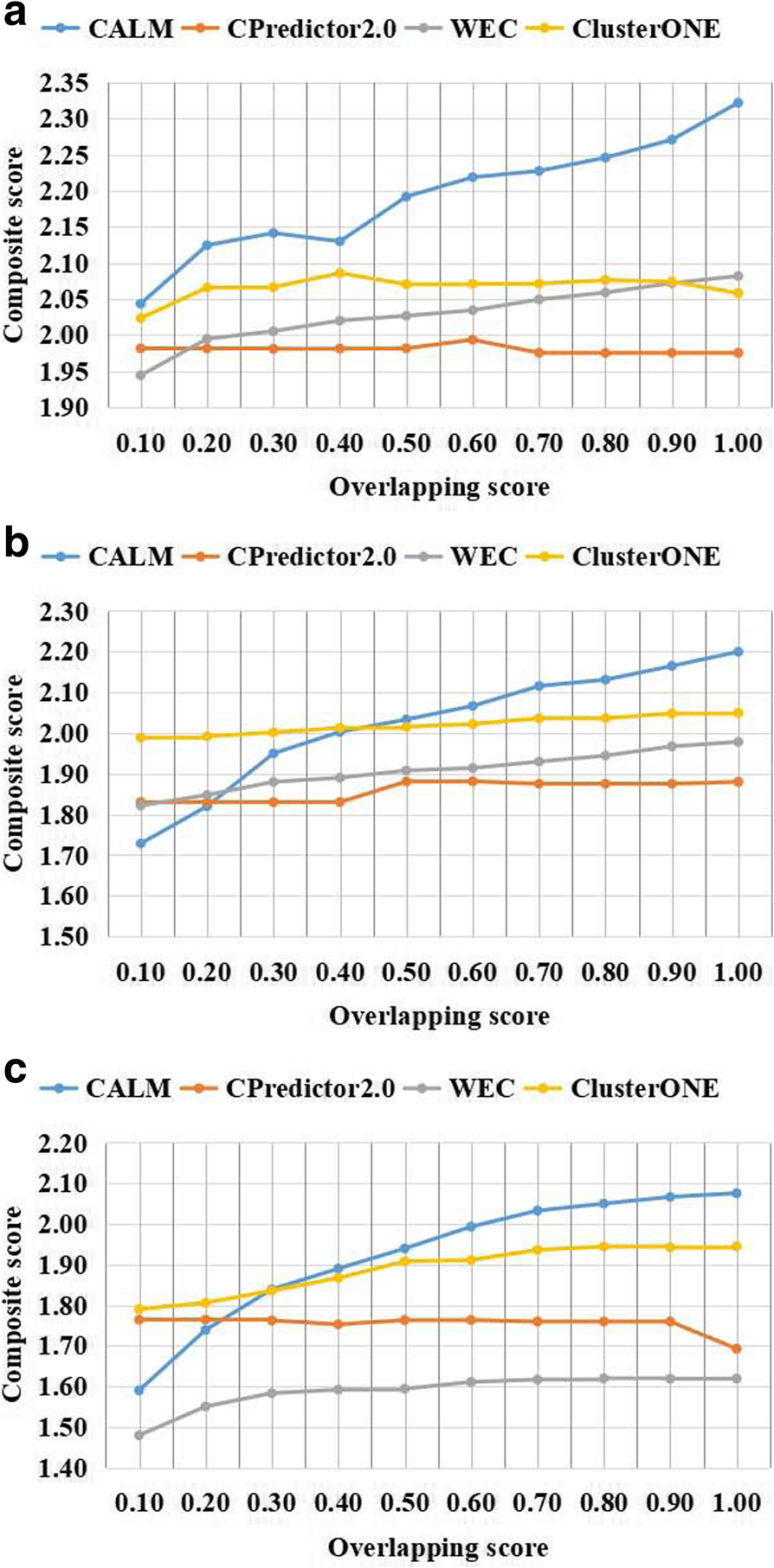
Composite score using CYC2008 as benchmark with respect to various overlapping score thresholds. Comparison of the composite score of CALM and other three the state-of-the-art methods from different weighted network with respect to different overlapping scores threshold (from 0.1 to 1 with 0.1 increment). Various PPI datasets include **a** Collins et al., **b** Gavin et al., **c** Krogan core et al. The value of the composite score include ACC, Fraction, and MMR

**Fig. 4:**
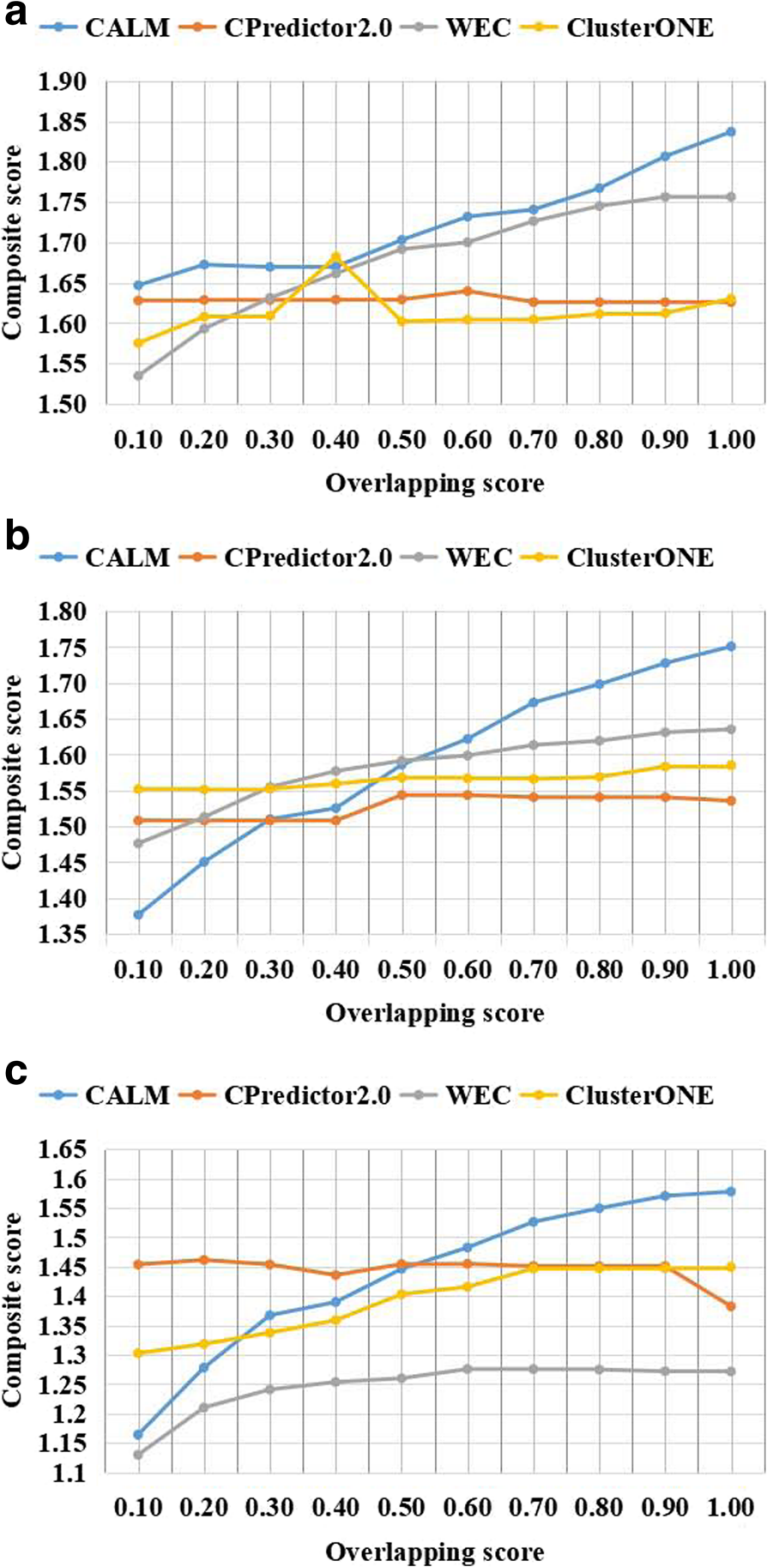
Composite score using NewMIPS as benchmark with respect to various overlapping score thresholds. Comparison of the composite score of CALM and other three the state-of-the-art methods from weighted network with respect to different overlapping scores threshold (from 0.1 to 1 with 0.1 increment). Various PPI datasets include **a** Collins et al., **b** Gavin et al., **c** Krogan core et al. The value of the composite score include ACC, Fraction, and MMR

CALM method includes one adjustable parameter that need be optimized, named *OS*. To understand how the value of *OS* influences the composite score, we first test the effect of using different overlapping score *OS* values for protein complex prediction, and we also carried out experiments on three datasets with *OS* varying from 0.1 to 1.0 and calculated the composite score. The results for the protein complexes are detected from the three weighted PPI networks of the yeast Saccharomyces cerevisiae are shown in Table 1. The performance is evaluated by the composite scores, which are calculated using CYC2008 and NewMIPS as the benchmark protein complexes. The comparison results with respect to different overlapping score thresholds *OS* are shown in Figs. 3 and 4. Note that the results of CYC2008 and NewMIPS are shown separately.

Experimentation with different parameter values are performed to select the suitable parameters for CALM. Examination of Figs. 3 and 4 clearly shows the suitable parameters for CALM, the composite scores show similar trends in all datasets, with the composite score increasing with the increase in the overlapping score threshold *OS*. Overall, we find that CALM shows a competitive performance when *OS*=1.0. To avoid evaluation bias and overestimation of the performance, we do not tune the parameter to a particular dataset, and set *OS* to 1.0 as the default value in the following experiments.

It can be seen from Figs. 3a and 4a, that the composite score of CALM is always higher than other methods. It could be seen from Fig. 3b and c, that when the overlapping score is in the 0.1-0.4 range, the composite score from CALM is slightly lower than the scores obtained using other methods. However, when the overlapping score is in the 0.4-1.0 range, the composite score from CALM is clearly higher that those of the other methods. It can be seen from Fig. 4b and c, that when the overlapping score is in the 0.1-0.5 range, the composite score from CALM is slightly lower the those for the other methods. However, when the overlapping score is in the 0.6-1.0 range, the composite score from CALM is clearly higher than those obtained using other methods. WEC and CPredictor2.0 are insensitive to the selection of *OS*, because these method for identification PCs are based on not only topological informations but also other biological informations include functional annotations and gene expression profile. However, CALM and ClusterONE show that their composite scores are increasing as *OS* increases. It could be seen from the above comprehensive analysis, the experimental results show that CALM has a significant performance advantage over the other three competing methods in terms of the composite score in most cases. In summary, CALM shows relatively higher robustness to parameter choices.

For a fair comparison, all parameters in these compared methods are set as suggested by their authors or to the parameters corresponding to the best results. The parameters used and the rationale behind the choice of parameter values are described in the Additional file 1.

### Comparison with existing methods

**Fig. 5:**
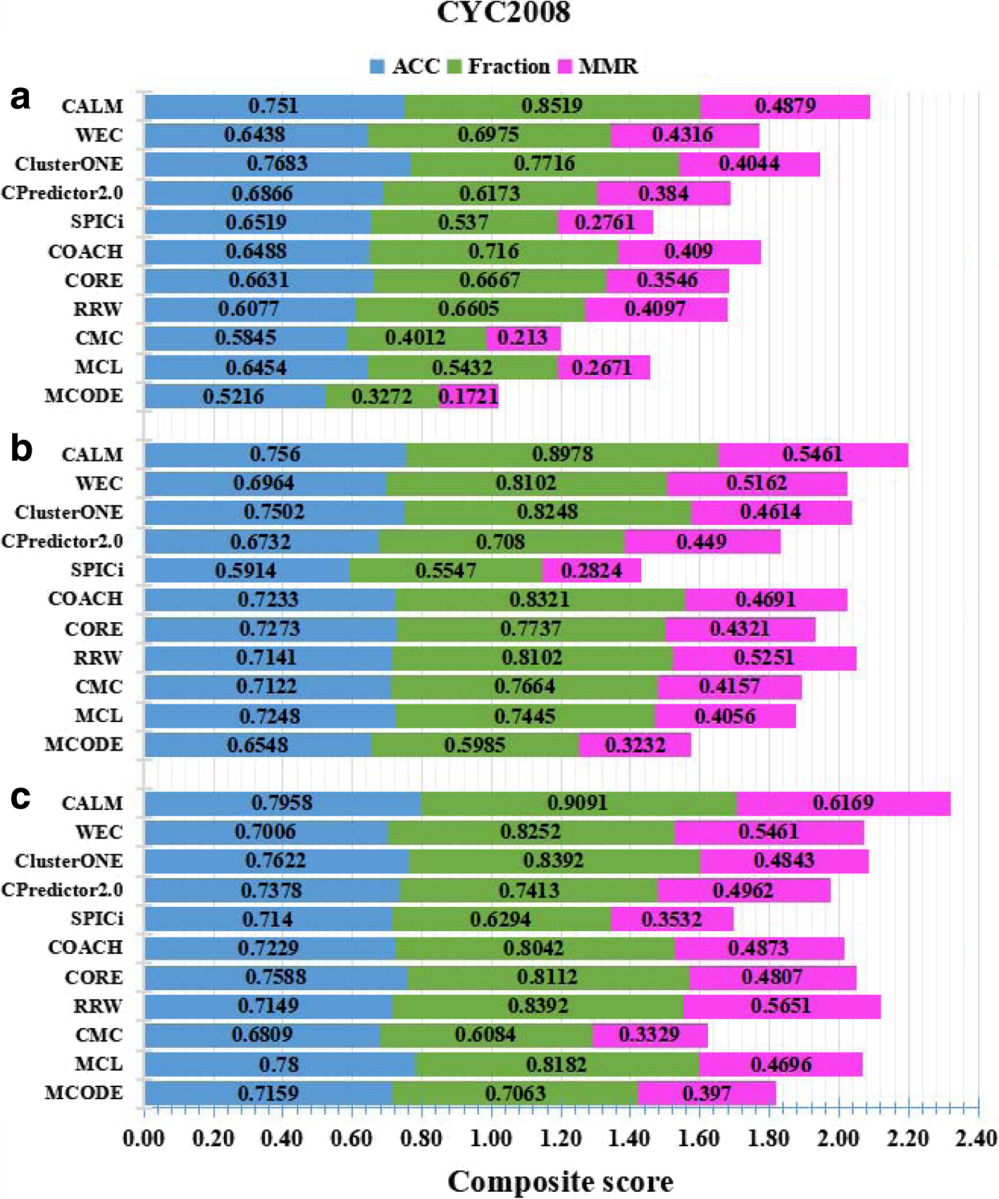
Prediction performance on three PPINs and CYC2008 is used as benchmark. The comparisons are in terms of the geometric accuracy (ACC), the fraction of reference complexes which are matched by at least one predicted cluster (Fraction), and the maximum matching ratio (MMR). Various PPI datasets include **a** Collins et al., **b** Gavin et al., **c** Krogan core et al. The total height height of each bar is the value of the composite scores of three metrics on a given network. Larger scores are better

**Fig. 6:**
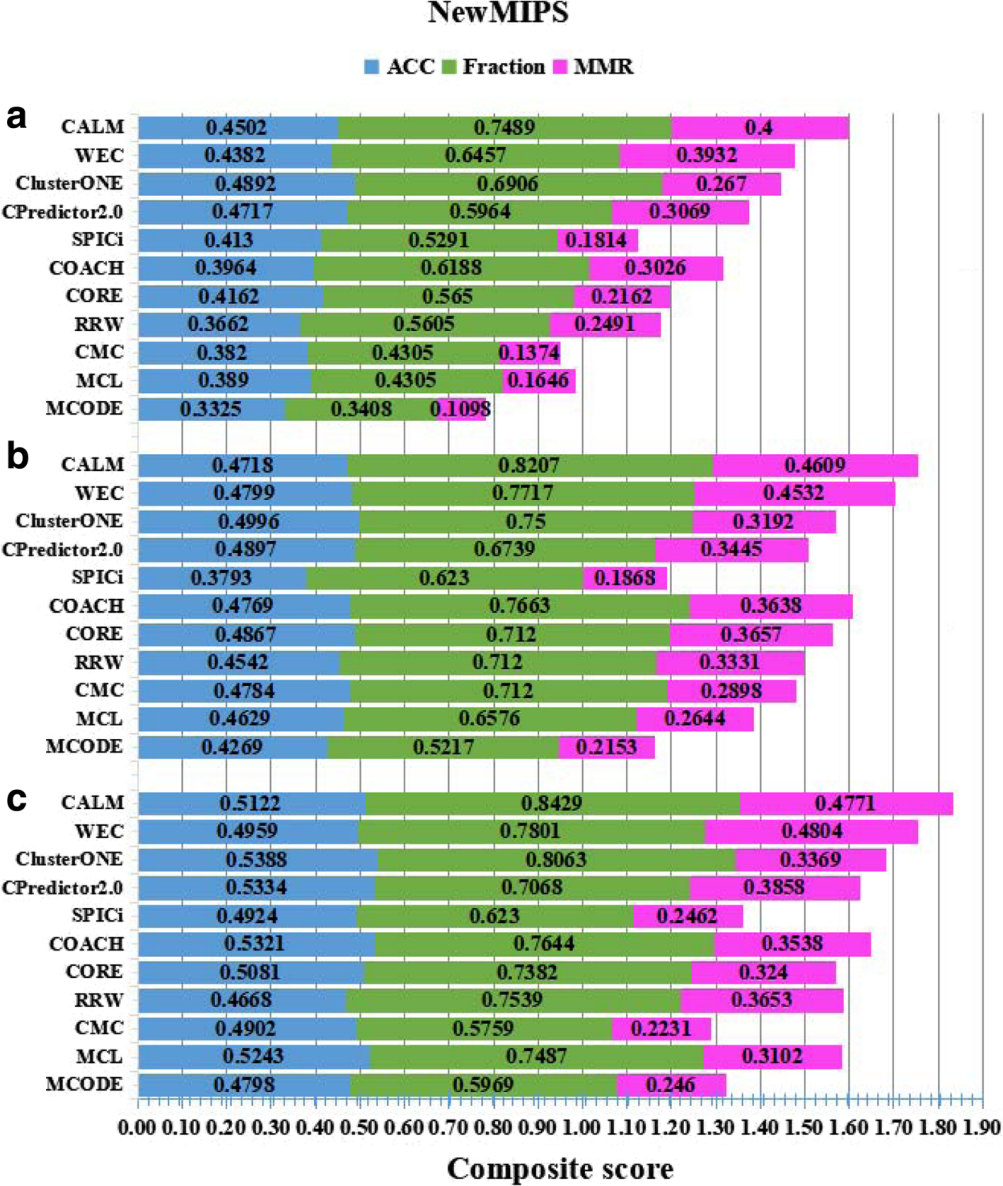
Prediction performance on three PPINs and NewMIPS is used as benchmark. The comparisons are in terms of the geometric accuracy (ACC), the fraction of reference complexes which are matched by at least one predicted cluster (Fraction), and the maximum matching ratio (MMR). Various PPI datasets include **a** Collins et al., **b** Gavin et al., **c** Krogan core et al. The total height height of each bar is the value of the composite scores of three metrics on a given network. Larger scores are better

CALM has been evaluated on three PPINs by taking into consideration NewMIPS and CYC2008 as benchmark datasets. The details of the experimental results are shown in Figs. 5 and 6. Furthermore, we compare CALM with ten existing state-of-the-art protein complex detection methods which include MCODE [14], MCL [2], COACH [34], CORE [35], CMC [12], CPredictor2.0 [18], RRW [15], SPICi [5], ClusterONE [24], and WEC [33]. Some of these (such as COACH and CORE) cannot handle weights of PPINs, and thus the weight is ignored. Here, for all compared methods, similar to CALM, we exclude complex candidates with the size of fewer than three proteins. The aforementioned weighted PPINs are used to detect the PCs. For CALM, we set the merging threshold *ω* as 1.0. We do not tune any parameters to a particular dataset, and all parameters of CALM are set to default or are computed automatically. The performances of these representative methods are evaluated by ACC, fraction and MMR. These comparison approaches are provided and used in Ref [24].

The experimental results obtained using CYC2008 dataset as benchmark are shown in Fig. 5. CALM achieves the highest fraction and MMR in three weighted PPINs. It is obvious that CALM is much better than other prediction methods in terms of fraction and MMR. For Fraction, It means that CALM could identify more PCs. For MMR, all other methods show obvious lower score than CALM, indicating CALM has better performance for the identification of overlapping complexes. Compared to other methods, CALM’s ACC is a slightly lower than the ACC of ClusterONE in the Collins datasets **(a)**. For the ACC, which consists of Sn and PPV, PPV tends to be lower if there are substantial overlaps among the detected PCs. A more in-depth analysis has been demonstrated by Nepusz et al. [24]. On the contrary, CALM achieves the highest fraction and MMR in all datasets, and obviously outperforms other methods. As shown in Fig. 5, the total height of each bar is the composite score of three metrics (ACC, fraction, MMR) for different methods on different PPINs. The higher score is better. Based on all experimental results obtained using the CYC2008 dataset and shown in Fig. 5, we could conclude that all comparison methods have different performance on different PPINs. Some of these are the state of art innovative approaches such as ClusterONE, WEC, and CPredictor2.0 developed in recent years. Nevertheless, the performance of CALM is more stable and robust for the three weighted PPINs used. Thus, CALM achieves an overall best performance among the eleven methods compared.

The results using NewMIPS as benchmark are illustrated in Fig. 6. The performances of all methods are basically consistent with Fig. 5. It is obvious that CALM dominates other methods in term of fraction and MMR. For ACC, all other methods shows an obvious instability, whereas CALM always stays at the second or third position in all methods, our ACC is very close to the best. In summary, the ACC of our method is slightly lower than the best result. Meanwhile, CALM is quite competitive and the best in terms of fraction and MMR in several PPINs. This means that CALM could identify more overlapping PCs. Similarly, we also compute the composite score by using NewMIPS as benchmark as shown in Fig. 6. Based on the result of the composite scores, CALM clearly outperforms the other comparison methods. All in all, comparing Figs. 5 and 6, we could conclude that for the PPINs (Collins, Gavin or Krogan), the performance with CYC2008 as reference set is better than NewMIPS as the reference set because the number of PCs is higher in NewMIPS than in CYC2008.

## Conclusion

In this paper, we develop a clustering method called CALM for PCs detection based on the core-attachment and local modularity structure from weighted PPI networks. It could be seen from the experimental results that CALM outperforms ten other state-of-the-art methods in term of three evaluation metrics. CALM considers many aspects about PPIN and PCs, including noise data, core-attachment structure, local modularity structure, overlapping PCs and various density PCs. Therefore, CALM could get a novel insight for predicted complexes in bioinformatics field. For this purpose, we first identify overlapping nodes and seed nodes according to the properties such as weight degree and node betweenness, and then we expand each cluster from each seed node based on the core-attachment structure. Furthermore, we generate candidate clusters by using seed selection and local and greedy search process. Note that each seed node in PPI network is extended only once. Finally, we merge and remove some candidate clusters, the rest of the candidate clusters are considered as PCs. In addition, CALM thoroughly considers two major limitations in PPINs, namely, incompleteness and high noise data. In conclusion, CALM outperforms the competing approaches and is capable of effectively detecting both overlapping PCs and varying density PCs. What’s more, we study some topological properties for the identification of overlapping nodes, which has not been researched before.

In the future, firstly, we are considering more efficient methods to improve the performance for the accuracy of the identified overlapping complexes. Secondly, it will be worthwhile to develop a measure for assessing the reliability of protein interactions and so that CALM could detect the PCs in unweighted PPI datasets. Thirdly, CALM could be applied in related fields such as the analysis of social networks.

## Additional file

Additional file 1 Predicting overlapping protein complexes based on core-attachment structure and a local modularity measure. (TEX 16 kb)

## Abbreviations

ACC: The geometric accuracy
CALM: Core-attachment and local modularity structure
ClusterONE: Clustering with overlapping neighborhood expansion
CMC: Clustering based on maximal cliques
CPM: Clique percolation method
Fraction: Fraction of matched complexes
G-N: Girvan and Newman
GO: Gene ontology
MCL: Markov cluster
MCODE: Molecular complex detection
MMR: The maximum matching ratio
PCs: Protein complexes
PPINs: Protein-protein interaction networks
PPV: Positive predictive value
RRW: Repeated random walks
SPICi: Speed and performance in clustering
Sn: Clustering-wise sensitivity
WEC: Weighted edge based clustering
